# Field of Attention for Instantaneous Object Recognition

**DOI:** 10.1371/journal.pone.0016343

**Published:** 2011-01-21

**Authors:** Jian-Gao Yao, Xin Gao, Hong-Mei Yan, Chao-Yi Li

**Affiliations:** 1 Key Laboratory for Neuroinformatics, Ministry of Education of China, University of Electronic Sciences and Technology, Chengdu, China; 2 Center for Life Sciences, Shanghai Institutes of Biological Sciences, Chinese Academy of Sciences, Shanghai, China; Center for Genomic Regulation, Spain

## Abstract

**Background:**

Instantaneous object discrimination and categorization are fundamental cognitive capacities performed with the guidance of visual attention. Visual attention enables selection of a salient object within a limited area of the visual field; we referred to as “field of attention” (FA). Though there is some evidence concerning the spatial extent of object recognition, the following questions still remain unknown: (a) how large is the FA for rapid object categorization, (b) how accuracy of attention is distributed over the FA, and (c) how fast complex objects can be categorized when presented against backgrounds formed by natural scenes.

**Methodology/Principal Findings:**

To answer these questions, we used a visual perceptual task in which subjects were asked to focus their attention on a point while being required to categorize briefly flashed (20 ms) photographs of natural scenes by indicating whether or not these contained an animal. By measuring the accuracy of categorization at different eccentricities from the fixation point, we were able to determine the spatial extent and the distribution of accuracy over the FA, as well as the speed of categorizing objects using stimulus onset asynchrony (SOA). Our results revealed that subjects are able to rapidly categorize complex natural images within about 0.1 s without eye movement, and showed that the FA for instantaneous image categorization covers a visual field extending 20°×24°, and accuracy was highest (>90%) at the center of FA and declined with increasing eccentricity.

**Conclusions/Significance:**

In conclusion, human beings are able to categorize complex natural images at a glance over a large extent of the visual field without eye movement.

## Introduction

Instantaneous object discrimination and categorization are fundamental cognitive behaviors and are of crucial importance for the survival of most animals, and human activity also relies on fast classification and identification of visual objects. Psychophysical experiments [1]–[6] and functional imaging studies on humans [7]–[9] and single unit recordings on non-human primates [10]–[12] have shown that humans and other primates can recognize objects very rapidly, even when these objects are presented in different size, color and rotation. Because only the central 2° of visual field (fovea) can produce sharp vision, it is generally believed that object recognition requires successive saccadic eye movements to bring objects of interest into fovea [13]. Although little is known about the object recognition in peripheral vision [14]–[16], our experience of everyday vision implies that we can rapidly and effortlessly recognize objects even when they suddenly occur at an unexpected peripheral location. The aim of the present study is to determine whether human being is able to recognize object instantaneously using peripheral vision without saccadic eye movement, and if so, how large is the field of attention (FA) for instantaneous object recognizing and how is recognizing accuracy distributed over the field. We used visual perceptional tasks in which subjects were asked to focus their attention to a point (fixation point, FP), and meanwhile simple letters or photographs of complicated natural scenes were briefly flashed at different eccentricities of the testing field. The subjects had to distinguish the letters and to categorize photographs of natural scenes within the field. Because of the high variability of the stimulus locations and the very short presentation time, subjects were obliged to spread attention equally across the entire testing field while their attention was directed to the FP. By measuring the accuracy rate over the field, we were able to determine the spatial extent and the sensitivity distribution of the FA for the letter discrimination and for image-categorization tasks.

## Materials and Methods

### Subjects

Six right-handed subjects were tested in the experiment. Ages of the subjects ranged from 24 to 26, all are undergraduates or postgraduates of University of Electronic Science and Technology of China. All subjects provided written informed consent and all research was approved by the Ethics and Human Participants in Research Committee, University of Electronic Sciences and Technology of China, Chengdu, China. In addition, all subjects reported normal color vision and normal or corrected-to- normal visual acuity. Each subject enrolled for about 5–6 daily sessions of 2.5–3 hours.

### Experimental setup

Subjects were seated in a dark room specially designed for psychophysical experiments. Ambient illumination was approximately 5 cd/m^2^. Stimuli were presented on a high-resolution color monitor (1,024×1,280 pixels, 3×8 bit RGB), connected to an EyeLink 2000 display computer. The refresh rate of the monitor was 100 Hz, permitting display times to be varied in steps of 10 ms. A chinrest was used. Viewing distance was approximately 57 cm, allowing a display of approximately 30°×40° of visual angle. During the trials, subjects were instructed to fixate on the FP at the display center, and an infrared eye tracker (Eyelink2000, SR Research Ltd.) was used to monitor the fixation of the eyes. If the gazing position of eyes deviated more than 1° from the FP, the trial was discarded and another trial was supplemented automatically.

### Training procedure

**Figure 1:**
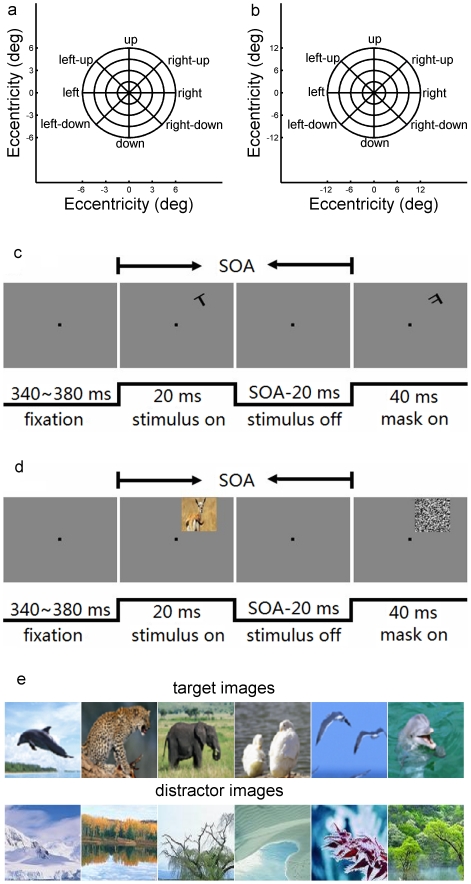
Experimental protocol. (**a, b**) Stimulus locations. The stimulus image was presented randomly at 33 possible locations which were distributed along eight radial axes, with five different eccentricities at each axis. The eccentricities used for rapid letter discrimination task (**a**) are 0°, 1.5°, 3°, 4.5° and 6°, and for rapid natural image categorization task (**b**) are 0°, 3°, 6°, 9° and 12°. (**c**, **d**) Schematic illustration of the experimental procedures. The tasks started with a fixation point (FP) on the center of the screen, 340∼380 ms before the onset of the stimulus. The stimulus was presented for 20 ms randomly at different locations. Then a blank interval of variable duration was set after termination of the stimulus, followed by a 40 ms-mask. SOA was calculated as 20 ms stimulus time plus duration of the blank interval. In letter discrimination task (**c**), the stimulus was a randomly rotated letter “T” or “L” (size 1°×1°), the mask was of a letter “F” at the same location as the stimulus. In natural image categorization task (**d**), the stimulus was an image of natural scenes (size 3°×3°), which could be a natural scene (without rotatation) with animal in it (target images) or without animal (distractor images); the mask is a noise image. (**e**) Samples of target images and distractor images. The total of the target images was 250, and that of distractor images was 240, both were taken from a commercially available CD.

The experiment required a training period, it usually took about 5 h for the subjects to coordinate their motor responses well enough to respond to the task. The effective presentation time of a stimulus was determined not by the physical presentation time (20 ms) but by the time between onset of the stimulus and onset of the mask, or stimulus onset asynchrony (SOA) (see Fig. 1c and d). The onset of the mask limited visual persistence of the after-image. The SOA started at 500 ms, and then decreased when the performance correctness (accuracy) of the task exceeded 90%. The training procedure was terminated when the subject's performance had stabilized and SOA could not decrease further. The final SOA ranged from 70 to 110 ms for different subjects (Table 1).

**Table 1:** SOA values for the six subjects.

Subjects	SOA (ms) for letter discrimination task	SOA (ms) for image categorization task
YJG	80	90
CYP	90	90
GX	110	100
CHY	100	110
ZT	100	110
LC	90	90
Mean	95	98

### Experimental paradigm

**Figure 2:**
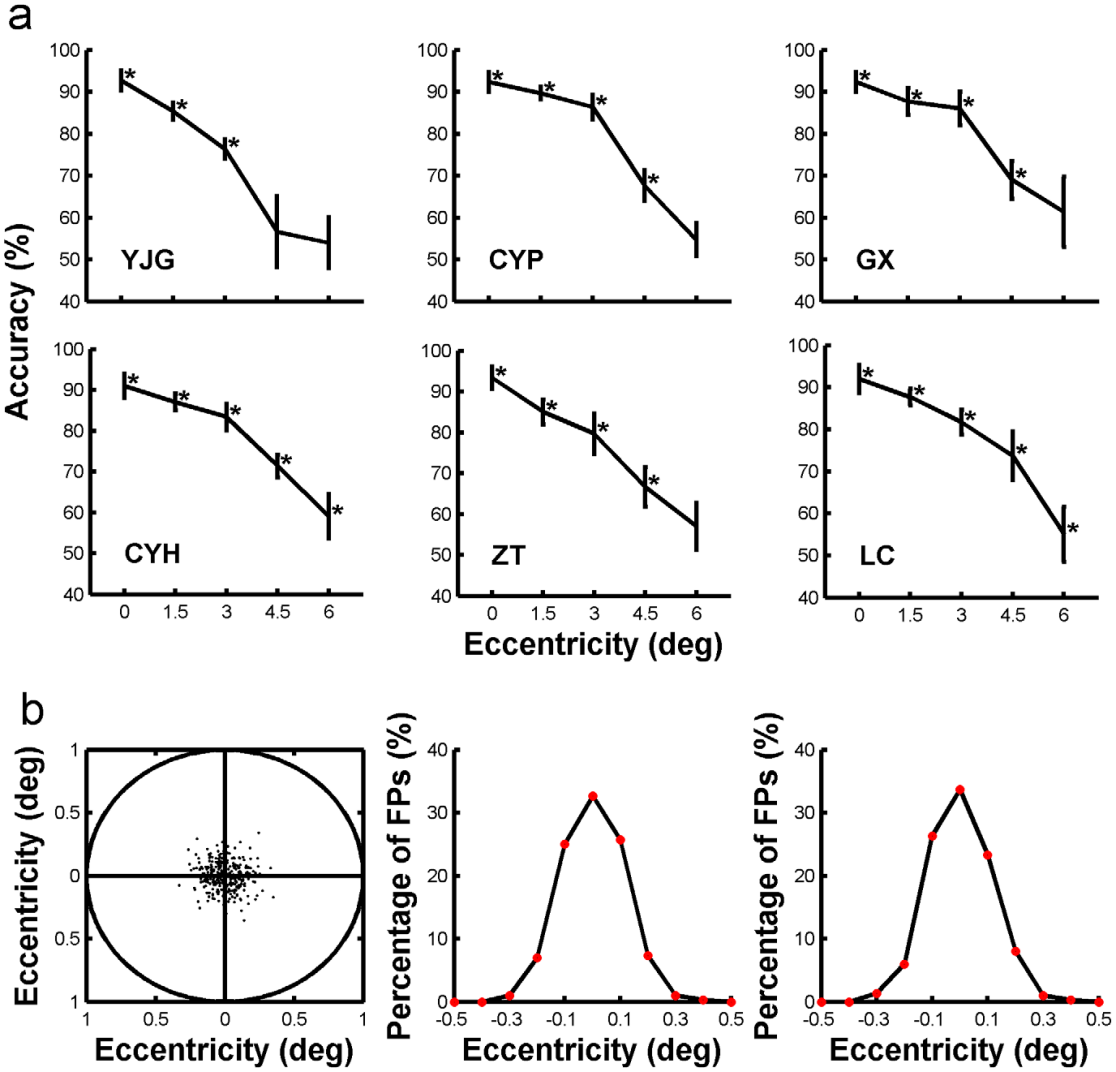
Performances of six subjects in rapid letter discrimination task. (a) The horizontal axis represents eccentricity (deg). The vertical axis represents accuracy rate (%, M±SD). Sign* means significant difference (p<0.01). (b) An example of the real fixation positions during the task. In the left subgraph, each single point represents the real fixation position in one trial, the circle outside covers a range of 1°eccentricity centered at the FP. The middle sub graph shows the distribution of fixation position along the X-axis, and the right sub graph, the distribution of fixation position along the Y-axis; the horizontal axis represents eccentricity (deg), and the vertical axis represents the relative number of fixation locations, both peaked at the central fixation point.

While the subjects were focusing their attention to the FP on the center of a monitor screen, short flashed (20 ms duration) images of letters or natural scenes were presented randomly at 33 possible locations distributed along eight radial directions at 5 different eccentricities (Fig. 1a, b). The experiment consisted of two recognition tasks. (a) *Instantaneous letter discrimination:* the subjects were asked to discriminate letter “T” from “L” (size 1°×1°, randomly rotated) instantaneously at different locations (centered at 0°, 1.5°, 3.0°, 4.5°and 6.0° eccentricities) (Fig. 1a); the task was terminated by presenting a perceptual mask (letter “F”, 40 ms duration) at the same location after a time interval (“stimulus-off time”, Fig. 1c). (b) *Instantaneous natural image categorization*: the task required the observer to categorize photographs of natural scenes (3°×3° size) by answering whether or not they contained animals. The stimulus was presented randomly at different locations centered at 0°, 3.0°, 6.0°, 9.0° and 12.0° eccentricities (Fig. 1b) and was masked by a noise image after an interval (“stimulus-off time”, Fig. 1d). In each task, subjects were asked to respond as soon as possible by pressing one of two keys on the keyboard, one key was hit when they saw letter ‘T’ or the animal-containing image (“target images”, Fig. 1e), the other was hit when they saw letter ‘L’ or the non-animal image (“distractor images”, Fig. 1e). Each task included 1500 trials that were distributed at 5 eccentricities with 6 blocks for each eccentricity. It is important to note that because the position of the flashed photograph was random and highly variable over a broad field, subjects could not direct their attention in advance to a particular location, and were obliged to spread attention across the entire testing field. In addition, the very short presentation time did not allow the subject's eyes to make saccadic movement to the target. The stability of eye position was further ensured by the control of eye movements (Fig. 2b).

### Data base

The stimulus photographs used in the instantaneous natural image categorization task were complex color scenes taken from a commercially available CD-ROM library. Two hundred and fifty images were selected as target images, they are pictures of natural scenes containing one or more animals, including mammals, birds, fish, insects, and so on. The other two hundred and forty pictures were selected as distractor images, they are pictures of various natural scenes without animal. Some examples of the target and distractor images are shown in Fig. 1e.

## Results

### Field of attention (FA) for instantaneous letter discrimination

To show the relationship between the accuracy for letter discrimination and eccentricity of the target, the accuracy at each of the five eccentricities is expressed as the mean values of the 8 radial directions at the same eccentricity circle. The accuracy-eccentricity distribution curves for the six subjects are summarized in Fig. 2a. As expected, discrimination accuracy drops with increasing eccentricity. For all of the observers, the accuracy was over 90% for the centrally (0° eccentricity) presented letters, it decreased to about 80% at 3–4° eccentricity, and to 55–60% (a level just above chance) at 6° eccentricity (two-paired one-sample t-test, p>0.01). To ensure that there were no significant eye movements occurring during the entire fixation and testing period, the real fixation positions of the eyes were monitored with an infrared eye tracker (Eyelink 2000). The left subgraph in Fig. 2b shows an example of the eye movement recordings. The points represent distribution of the real fixation positions during the task, and each single point represents the real fixation position in one trial. The circle outside the points indicates a range of 1°visual angle. The curves in the middle and right subgraphs illustrate respectively the distribution of the relative number (%) of the real fixation points over the horizontal and vertical axes; both reveal a normal distribution, with a peak at the assigned FP (0° eccentricity) and a dynamic range of about 0.3° (radius) in both axes.

**Figure 3:**
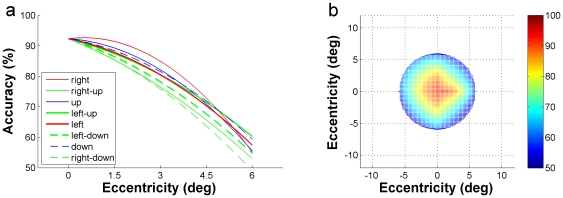
Mean results of accuracy-eccentricity distribution for rapid letter discrimination. (a) The eight curves marked by different colors are 2-order polynomial fitting of the average performance of the six subjects, representing respectively the accuracy-eccentricity distribution of each of the eight directions. (b) The average FA for rapid letter discrimination for the six subjects, accuracy at each of the 33 stimulus locations is presented in different colors as is shown in the right column. 0° in the coordinate represents the location of the FP.

In Fig. 3a, the eight curves (marked by different colors) represent respectively the accuracy-eccentricity distribution of each of the eight directions. All were obtained by averaging the data of the 6 subjects at each of the five eccentricity positions for a given direction. The two-dimensional plot in Fig. 3b is deduced from Fig. 3a, in which the accuracy-eccentricity distributions of the eight directions are presented in the stimulus-positions coordinate (see Fig. 1a) and are represented by variable colors (right column). This two-dimensional plot is defined as FA for instantaneous letter discrimination. It means that while the subjects are focusing their attention to a point, a letter can be discriminated instantaneously within this range with a certain accuracy. The shape of the FA for letter discrimination exhibits a 12°×12° rhombus centered at the fixation point, characterizing by a larger extent in the horizontal and vertical directions then in the oblique directions.

### Field of attention for instantaneous image categorization

**Figure 4:**
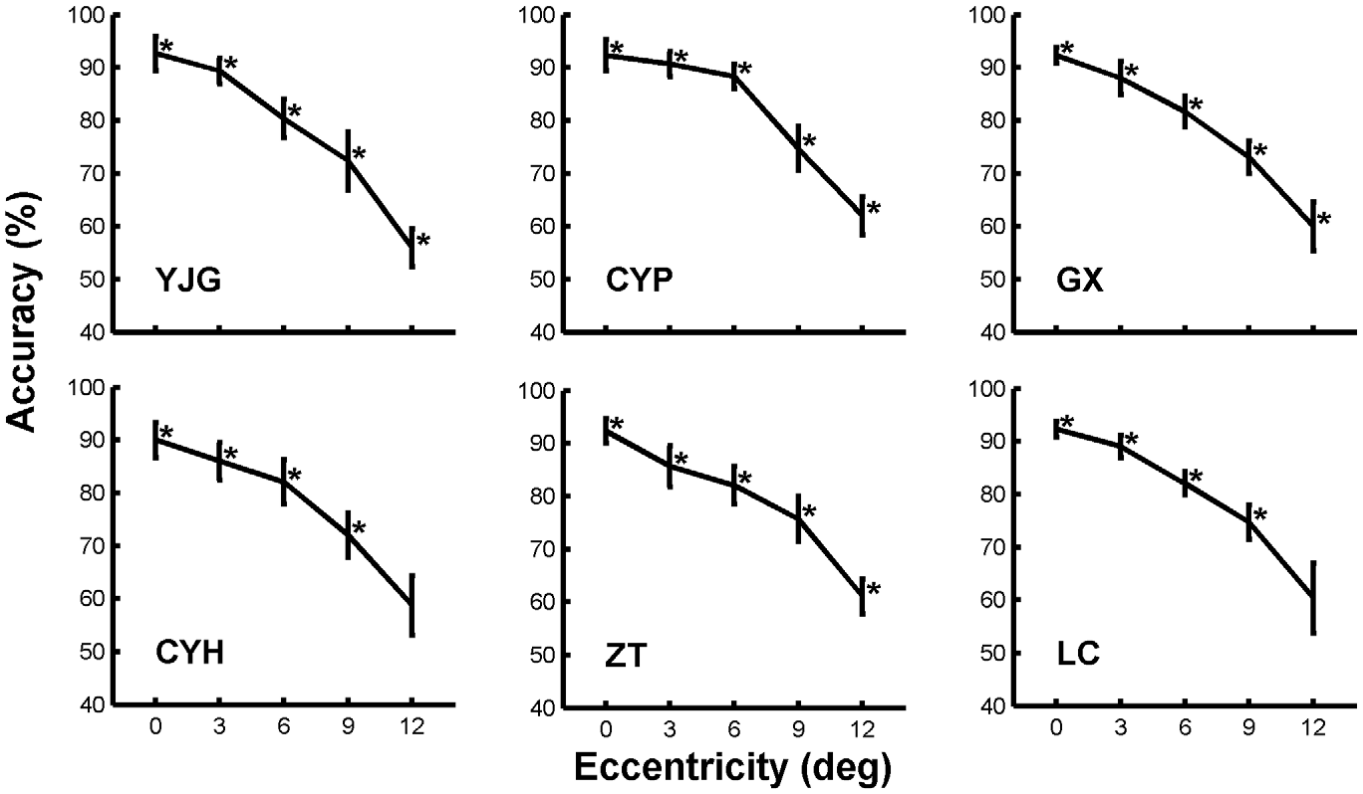
The accuracy-eccentricity distribution curves for rapid natural image categorization of the six subjects. Others are the same as in Fig. 2a.

For image categorization task, the subjects were required to categorize photographs of natural scenes by answering whether or not they contained animals. The accuracy-eccentricity distribution curves of the six subjects for rapid natural image categorization task are shown in Fig. 4. As it has been shown for letter discrimination task, for all of the observers, the accuracy for natural image categorization declined with increasing eccentricity, it was highest (≥90%) at the focusing center (0° eccentricity) and decreased to about 80% at 6°,75% at 9°, and 55–60% at 12°.

**Figure 5:**
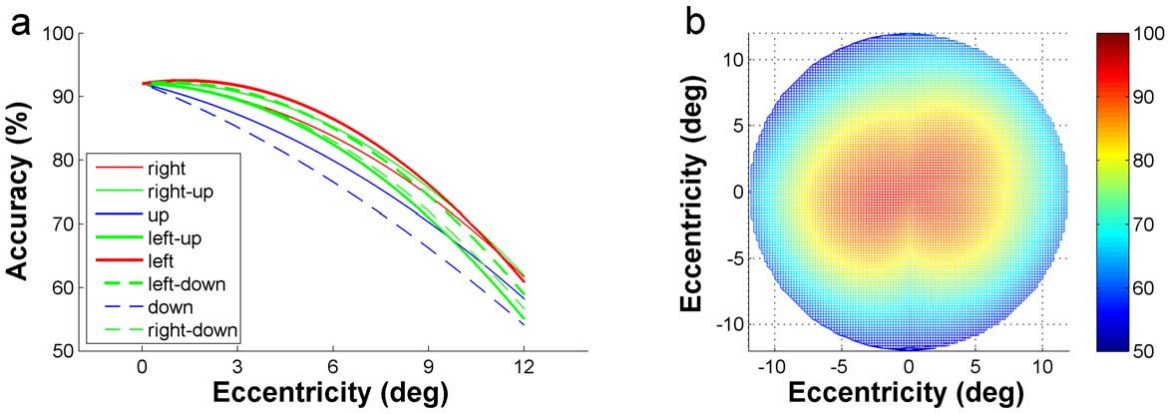
Mean results of accuracy-eccentricity distribution for rapid natural image categorization. (a) The accuracy-eccentricity distribution curves of the six subjects for image categorization task at the eight radial directions. (b) The average FA for rapid image categorization for the six subjects, accuracy at each of the 33 stimulus locations is presented in different colors.

Fig. 5a shows the accuracy-eccentricity distribution curves for image categorization task at the eight radial directions. Each curve was the average of the data of the 6 subjects. Fig. 5b was deduced from Fig. 5a, representing the FA for instantaneous image categorization for the six subjects. The accuracy distribution in the FA indicates that while attending to a given point in the visual field, a complicated natural image can be categorized instantaneously with a high accuracy (≥90%) at the attended point (0° eccentricity), a considerably high accuracy (≥80%) within a range of 6° height ×8° width eccentricity, and the accuracy is well above the chance level until 10° (height) ×12° (width) eccentricity. The shape of the FA for instantaneous image categorization is thus a 20°×24° ellipse (centered at the fixation point), with an inflection at both sides of the vertical midline.

### Processing time for letter-discrimination task and image-categorization task

The stabilized SOA for both letter-discrimination and image categorization tasks ranged from 70 to 110 ms for different subjects (Table 1), no significant differences were seen between the two types of recognition tasks.

**Figure 6:**
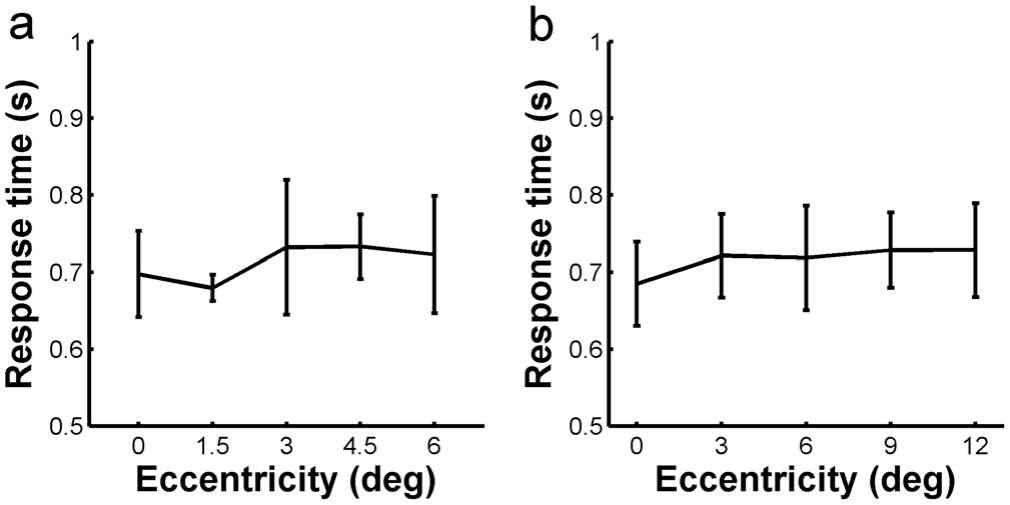
Reaction times of the subjects in performing letter discrimination (a) and natural image categorization (b) tasks. The x-axis represents eccentricity in the FA, 0° eccentricity represents the center of FA. The y-axis shows the reaction times, the values are the mean of the six subjects, and the bars represent ±SD.

In Fig. 6 is shown the reaction time (mean±SD) of the six subjects in performing the two types of tasks. The results illustrate that the reaction times for letter discrimination task (a) and for image categorization task (b) were all in the same range, both were about 700 ms on the average, no matter the task is simple (letter discrimination) or complex (natural scene categorization), or the object is presented in the center of FA (0° eccentricity) or in its periphery (6° eccentricity for letter discrimination, 12° eccentricity for image categorization).

## Discussion

### Field of attention and accuracy distribution

In the natural environment, there is far more perceptual information than that we can effectively process. To cope with this information overload, visual attention allows people to select the limited information that is most relevant to ongoing behavior and to ignore the irrelevant or interfering information. This selection of attention can be conceived of as a mental spotlight [17] that can be shifted to relevant locations and facilitates the processing of information within the range of attention. Stimuli falling within the beam of attention are enhanced and discriminated more rapidly and accurately than stimuli at unattended locations. The spotlight metaphor is useful for understanding how attention is deployed across space. However, this metaphor needs revision because later reports demonstrated spatial spread of attention that follows a gradient with decreased effects of attention with increased eccentricity from its focus [18]–[21].

With regards to high-level perception such as rapid visual categorization of novel natural scenes, some investigators believe that it requires very little or no focal attention [5] and that it can be achieved even at the peripheral visual field where the images were centered at 70° beyond the focusing center of attention [16]. In the present study, we used similar experimental paradigm to explore the effects of visual attention on categorization of natural scenes, with emphasis on the spatial extent and the processing time of the attention effects. Our results demonstrated that while attending to a point, the natural scenes can be categorized rapidly within a certain range of visual space, and the accuracy for the natural scene categorization was high (≥90%) exclusively at the focusing center and declined with increasing eccentricity. We defined the field of attention (FA) for instantaneous image categorization as the visual space within which the observers can rapidly categorize objects with accuracy above the chance level (55–60%). The results of the six subjects illustrate that, without exploratory eye movements, the FA for natural image categorization covers a 20° height ×24° width visual field (centered at the fixation point); no hemispheric specialization is seen from the shape of FA. It is concluded that accurate categorization of natural images does require focal attention, but the peripheral FA within 10°×12° eccentricity may also categorize natural scenes to a certain degrees.

Despite the fact that the acuity of attention is increasingly coarser towards the periphery of FA, the peripheral attention, however, may play crucial roles in searching objects and in fine adjustment of attention focus. During the course of visual searching, observers may first use the relatively coarse but rapid peripheral attention to find potential relevant targets, such as to monitor stop signs, traffic lights, and other cars during driving, and then, the centripetal distribution of accuracy may provide a cue for fine adjustment of attention based on a perceptional focusing process, that makes a perceptional uncertain object to become certain by shifting the focal point of attention along the ascending distribution of accuracy.

As to the difference in the extent of FA for natural image categorization and for letter discrimination, the most probable explanations are that, first, the two types of FAs have different biological significances, and second, they are underlied by different levels of cortical mechanisms.

### Processing time for natural image categorization

Rapid perception has mostly been reported for basic features of objects, such as intensity [22]–[23], color [23], [24], line orientation [25], [26], size [27] and direction of motion [28], [29]. These visual features were generally described as “preattentive”, because these tasks can be completed with very little attentional effort [30]. Preattentive processing can help to rapidly draw the focus of attention to a target with a unique visual feature [31].

To recognize complex natural image or scenes appears to be instantaneous, but measuring the visual processing time accurately seems to be not readily soluble. Few attempts were made by using event-related potentials (ERPs) and reaction time [32]. By measuring ERPs it is possible to gain more insight into the exact time-course and the possible neural locus of the effects of spatial attention. Martínez et al. [33] investigated the cortical mechanisms of visual spatial attention while subjects discriminated patterned targets within distractor arrays. They found that ERPs of striate cortex occurred at 50–55 ms, and the earliest facilitation of attended signals was observed in extrastriate visual areas at 70–75 ms. Van Voorhis and Hillyard [34] found that the P1 component of ERP had a greater positive amplitude when the target was presented in the attended field, they also observed a decrement in the P1 amplitude which occurred as early as 65 ms when the target appeared in the unattended visual field. Mangun et al. [35] concluded on the basis of current-source density analyses that the P1 component of ERPs is generated in extrastriate areas. Thorpe et al. [6] used a categorization task (distinguishing the presence of an animal in a natural scene) to analyze ERPs of the subjects. They found a frontal negativity specific to no-go trials that developed roughly 150 ms after the stimulus onset. We used similar categorization task in the present study, the subjects were required to distinguish the presence of animal in the rapidly flashed (20 ms) photographs. Because the stimuli were shortly presented and the after image has been removed by the mask after a time interval, the minimum SOA value of the subjects may provides a more reliable perceptional indication in determining the processing time. For the six subjects we have tested, the SOA for the natural image categorization task ranged from 70 to 110 ms, with a mean of 98 ms for the sample of subjects (Table 1). Comparing with the ERP studies mentioned above, our results showed that the processing time estimated by the SOA is longer than that determined by the ERPs in the striate cortex and the extrastriate cortex. The difference might be attributed to the fact that all the studies conducted in the early stages of the visual cortex used relatively simple targets (contrast patterns of bars or circles), the latency of the ERPs thus obtained might not reflect the processing time needed for performing complex categorization tasks. On the other hand, comparing with the study of Thorpe et al. [6], the frontal negativity related to the complex natural image categorization task is considerably longer than the SOA we observed using similar tasks. As the authors explained, this long-latency component of ERP was specific to no-go trials and was observed at frontal sites, it may reflect frontal inhibition of the motor response on distractor trials. It is also most probably that the 150 ms latency may involve some higher functions of the brain, such as decision making and/or initiation of motor control. Although how the human visual system can categorize complex images from the natural environment in such a short time has remained a challenge, the fact that the processing time (determined by SOA) needed for complex natural image categorization (mean 98 ms) is almost as short as that needed for simple letter discrimination (mean 95 ms) may support the view that spatial attention acts at early stages of visual processing by enhancing perceptual sensitivity [36]. Similar conclusion can be drawn from the approximate values of reaction time needed for performing these two types of tasks (Fig. 6).
